# Promising Functional Readouts of Immunity in a Blood-Stage Malaria Vaccine Trial

**DOI:** 10.1371/journal.pmed.0020380

**Published:** 2005-11-08

**Authors:** Brendan S Crabb, James G Beeson

## Abstract

The authors discuss results from an early trial of a vaccine based on *Plasmodium* MSP-3 protein reported by Pierre Druilhe and colleagues.

Malaria remains one of the world's most pressing health problems. For many populations in tropical countries, the disease is a leading cause of mortality and morbidity, and more broadly constitutes a major impediment to development on a number of fronts [1]. The few tools presently available for control of malaria are largely limited to insecticide-treated bed nets and treatment of clinical episodes with antimalarial drugs. An effective malaria vaccine would greatly augment these control measures, and is probably essential if the global burden of malaria is to be markedly and sustainably reduced. The absence of a vaccine against malaria reflects the many deficiencies in our current understanding of the mechanisms and targets of immunity to malaria. Correlates of immunity to blood-stage infection are unclear, as is the nature of immune responses that should be induced by candidate vaccines.

## Challenges in Selecting Suitable Vaccine Candidates

There are many reasons why identifying the key mediators of immunity has been so challenging. Plasmodium falciparum, the major cause of malaria, is a complex organism presenting numerous antigens that could feasibly be targets of protective responses. Moreover, antigens are often polymorphic, and in some cases, they exhibit clonal variation through differential expression of multi-gene family members. During blood-stage infection, when disease occurs, P. falciparum resides in erythrocytes, cells that lack major histocompatibility complex molecules on their surface and which are therefore not directly targeted by T lymphocytes. The process of erythrocyte invasion is complex and involves many receptor–ligand interactions with a significant degree of redundancy; blocking one ligand can be circumvented by the use of others [2]. In addition, expression and correct folding of recombinant P. falciparum antigens has also proved a significant roadblock.

## MSP-3—An Encouraging Vaccine Target

Despite these challenges, several antigens expressed by merozoites, the extracellular form of the parasite that infects erythrocytes, have emerged as promising vaccine candidates. Pierre Druilhe and colleagues now present encouraging findings from a phase I trial of merozoite surface protein (MSP) 3 [3]. A number of studies have supported the potential development of MSP-3 as a vaccine since its discovery [4], including associations between MSP-3 antibodies and acquired immunity in population studies (see references cited in [3]). The vaccine is based on a highly conserved region of the protein, and is produced as a synthetic peptide, incorporating both T and B cell epitopes. Immunization of naïve volunteers was performed with two different adjuvants, Montanide ISA720 and alum, and with different antigen doses.

## Testing for Functional Immunity

Besides standard evaluation of safety and immunogenicity of the vaccine, Druilhe and colleagues also demonstrated that vaccine recipients who seroconverted had substantial parasite growth inhibitory activity in antibody-dependent cellular inhibition (ADCI) assays. In these functional assays, serum antibodies are incubated with parasites, together with monocytes, in vitro (the monocytes are necessary for parasite growth inhibition, but their precise role is not understood). Some 60% of individuals who received the three-dose regimen generated antibodies that recognize the native MSP-3 protein, and there was a strong association between MSP-3 antibody positivity and possession of ADCI activity. This strong association is particularly encouraging, and supports the premise that ADCI activity is antigen specific. In this regard, it is interesting that MSP-3 has emerged as just one member of the family of merozoite proteins that can loosely be regarded as “MSP-3–like” proteins, all of which are encoded in a gene cluster on Chromosome 10 [5]. Druilhe and colleagues suggest that MSP-3 family members, such as MSP-6, possess cross-reactive ADCI epitopes; therefore, antibodies elicited by the vaccine probably have multiple parasite targets. As MSP-3 is not essential to blood-stage development of the parasite, at least in vitro [6], cross-reactivity of protective antibodies induced by the MSP-3 vaccine with other targets is highly desirable. To address this issue, the activity and specificity of the vaccine-induced response could be explored further through the use of an MSP-3 “knockout” parasite line [6].

The researchers also used a novel approach to demonstrate antimalarial activity of vaccine-induced antibodies in vivo. Severely immunocompromised mice grafted with human blood cells sustain experimental infections with P. falciparum (in contrast to normal mice, which are resistant to P. falciparum infection). Injection of human monocytes from nonvaccinated donors and of antibodies from seropositive vaccine recipients led to clearance of parasitemia in this humanized mouse model, analogous to results achieved with in vitro ADCI assays. While the relevance of this model to immunity in human infections remains unclear, these results provide another positive indicator of an antiparasitic effect for the vaccine-induced MSP-3 antibodies.

Significantly, parasite inhibitory activity in ADCI assays was still detected among many volunteers at 12 months postvaccination, particularly among those vaccinated with alum as the adjuvant. The vaccine also induced IFNγ T cell responses, and this type of response has been associated with protective immunity in studies of natural and experimental infection in humans [7,8].

## Moving Forward

It is becoming increasingly clear that functional assays to study vaccine-induced responses are essential. Examining seroconversion by enzyme-linked immunosorbent assay or immunofluorescence using whole parasites is not sufficiently informative, and several recent studies have demonstrated limitations of standard assays. For example, antibodies to MSP-1 do not necessarily inhibit P. falciparum replication, and worse, some antibodies appear able to block the action of inhibitory antibodies [9]. A longitudinal study in Kenya found that antibodies to recombinant MSP-1 were not associated with protective immunity, whereas MSP-1–specific growth inhibitory antibodies were predictive [10].

As intimated above, a particular strength of the MSP-3 trial is that functional assays were utilized to assess the quality of the antibody response produced. However, it remains unclear if ADCI is a major mechanism of parasite clearance capable of conferring protective immunity in exposed individuals. Such studies have been hampered somewhat because, like most functional assays, ADCI assays are technically challenging. Moreover, immune individuals have also been shown to possess inhibitory antibodies that do not require cooperation with monocytes; rather, they directly inhibit erythrocyte invasion and parasite replication [11,12]. Ultimately, the protective effect of MSP-3–specific antibodies and ADCI needs to be evaluated in an efficacy-based clinical trial of this vaccine.

## Abbreviations

ADCI: antibody-dependent cellular inhibition
MSP: merozoite surface protein
